# A scoping review of neurodegenerative manifestations in explainable digital phenotyping

**DOI:** 10.1038/s41531-023-00494-0

**Published:** 2023-03-30

**Authors:** Hessa Alfalahi, Sofia B. Dias, Ahsan H. Khandoker, Kallol Ray Chaudhuri, Leontios J. Hadjileontiadis

**Affiliations:** 1 Department of Biomedical Engineering, Khalifa University of Science and Technology, Abu Dhabi, United Arab Emirates; 2 Healthcare Engineering Innovation Center (HEIC), Khalifa University of Science and Technology, Abu Dhabi, United Arab Emirates; 3 CIPER, Faculdade de Motricidade Humana, University of Lisbon, Lisbon, Portugal; 4 Parkinson Foundation, International Center of Excellence, King’s College London, Denmark Hills, London, UK; 5 Department of Basic and Clinical Neurosciences, Institute of Psychiatry, Psychology and Neuroscience, King’s College London, De Crespigny Park, London, UK; 6 Department of Electrical and Computer Engineering, Aristotle University of Thessaloniki, Thessaloniki, Greece

**Keywords:** Neurological manifestations, Predictive medicine

## Abstract

Neurologists nowadays no longer view neurodegenerative diseases, like Parkinson’s and Alzheimer’s disease, as single entities, but rather as a spectrum of multifaceted symptoms with heterogeneous progression courses and treatment responses. The definition of the naturalistic behavioral repertoire of early neurodegenerative manifestations is still elusive, impeding early diagnosis and intervention. Central to this view is the role of artificial intelligence (AI) in reinforcing the depth of phenotypic information, thereby supporting the paradigm shift to precision medicine and personalized healthcare. This suggestion advocates the definition of disease subtypes in a new biomarker-supported nosology framework, yet without empirical consensus on standardization, reliability and interpretability. Although the well-defined neurodegenerative processes, linked to a triad of motor and non-motor preclinical symptoms, are detected by clinical intuition, we undertake an unbiased data-driven approach to identify different patterns of neuropathology distribution based on the naturalistic behavior data inherent to populations in-the-wild. We appraise the role of remote technologies in the definition of digital phenotyping specific to brain-, body- and social-level neurodegenerative subtle symptoms, emphasizing inter- and intra-patient variability powered by deep learning. As such, the present review endeavors to exploit digital technologies and AI to create disease-specific phenotypic explanations, facilitating the understanding of neurodegenerative diseases as “bio-psycho-social” conditions. Not only does this translational effort within explainable digital phenotyping foster the understanding of disease-induced traits, but it also enhances diagnostic and, eventually, treatment personalization.

## Introduction

In 2017, 18-month-long search log data were analyzed to identify people with neurodegenerative diseases^1^. In this bulge of fruitful data harnessed to infer disease-induced behavioral cues, there is currently no need for more data. Still, the focus needs to be directed to making sense of it in characterizing subjects’ behavior and naturalistic human traits^2^. Understanding and detecting subtle disruptions in behavior, in early-stage neurodegenerative diseases, is a complex problem. This complexity arises from the theoretically independent, yet practically connected levels of analysis, from genes to molecules, cells, circuits, and eventually to behavior^3^. Bridging the explanatory gap between these levels of analysis has become feasible with the advancement in computational modeling^4^. The latter provided the principles for synthesizing disparate pieces of evidence, in unprecedented ways, to formulate new conceptualizations on specific phenomena of neurodegeneration. Regardless of the operating level, the merging of computational models, powered by artificial intelligence (AI) and big data, and behavioral neuroscience is critical for the future of neurology, given its key role in the rational development of timely efficient diagnostic and nosological models and treatment decisions/strategies. So far, advances in research have blurred the boundaries and established redefinitions for diagnostic criteria in Parkinson’s disease (PD)^5,6^, and in Alzheimer’s disease (AD)^7^, in particular. However, clinical practice in neurodegenerative diseases and AI speaks two different languages, and an effort for a translation framework is yet to be made, thereby establishing a paradigm shift from data-based to knowledge-based models, with an emphasis on patient-specific phenotype^8^. This translation rests on behavioral neuroscience, fostered by advancements in digital technologies that allow the aggregation of real-life data.

Furthermore, from a clinical perspective, neurodegenerative diseases usually never present unified symptom profiles^9^. Despite decades of research, the dissatisfaction with the diagnostic criteria of neurological and psychiatric disorders that stems from the covert heterogeneity of symptoms continues to obscure timely diagnosis and treatment^10^. Reliance on sum scores and thresholds, for such inconsistent syndromes, impedes underpinning the neurobiological correlates of the symptoms per se, irrespectively of the diagnosis of the patient. The concept of comorbidities is of particular importance, which stems from the network approach that envisions the causal interplay between symptoms, which does not necessarily originate from the same neural substrate, but rather from complex dynamic interactions among them^11^. Dealing with this level of complexity demands superb tools that originate not only from sophisticated computational models but also from a mechanistic understanding of multiple levels of analysis^3^. Take, for instance, PD; although a movement disorder, it is quintessentially a neuropsychiatric disorder given that it is preceded by and accompanied by dementia, depressive and psychotic symptoms^12^. In light of this, this paper moves away from the traditional group-wise analysis. It attempts to establish new insights into the neurobiological, technical, and clinical implications of disturbed behavioral characteristics. In this way, adopting complex and dynamic theories can foster rigorous scientific practice, stemming from idiographic approaches toward patient-specific contexts^13,14^. We consequently suggest an urgent call for refinement of the assessment and intervention tools with complete consideration of the multifaceted nature of these disorders in a naturalistic and timely manner, by the adoption of behavioral quantification.

**Fig. 1:**
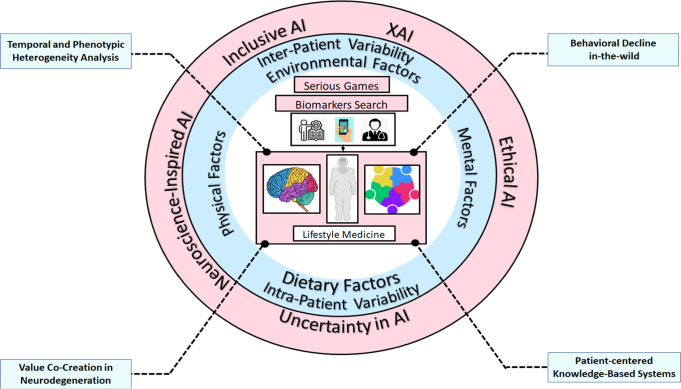
The taxonomy of explainable digital phenotyping of neurodegenerative diseases. The quantification of patient-specific subtle behavioral decline at the brain, body and social level is the microsystem of the taxonomy (white). The analysis of the interaction with environmental factors that aid in deciphering inter- and intra-patient variability is the mesosystem of the taxonomy (blue). The co-creation approach by neuroscientists, neurodegenerative disease specialists and bioengineers fosters the creation of a new AI landscape for personalized neurodegenerative disease characterization (pink).

It is now known that a continuum of non-specific neurodegenerative manifestations constitutes the prodromal stage of neurodegenerative diseases^15^. Neurology is, therefore, in dire need of translational research that accounts for the heterogeneous presentations of neurodegeneration to facilitate the development of targeted treatments. However, a uniform, multidisciplinary framework for deciphering this heterogeneity through robust computational analysis still needs to be developed. The co-existence of tremendous computational models for handling heterogeneous behavioral data resulted in the cultivation of data-driven approaches that lacks interpretability^16^. Besides that, a major challenge is to convert a huge amount of data into meaningful knowledge for neurologists. Arguing from a novel perspective, the merging of neurodegenerative background knowledge and the new knowledge inferred from naturalistic data is foreseen to establish a new definition of the prodromal phenotype^17^; a paradigm we introduce, called “explainable digital phenotyping” (see Fig. 1). The introduced taxonomy is motived by the ecological transition^18^ of neurodegenerative disease characterization, whereby the patients represent the microsystem level that includes the manifestations stemming from brain, body and social, behavioral decline. The mesosystem describes the influence of real-life contexts and environmental factors that result in phenotypic and temporal heterogeneity of neurodegenerative diseases. Lastly, we enclose in the taxonomy the role of neuroscientists, bioengineers and neurologists in cultivating new-generation AI systems within a co-creation macrosystem. Furthermore, given the interplay of genetics and environmental factors in inducing and modulating the neurodegenerative phenotype^19^, a precise definition of the latter is still elusive and should ideally be inferred from per-patient real-life characteristics and temporal behavior change, shifting the current paradigm from classification to forecasting. Thus, identifying the developmental process that leads to neurodegeneration becomes feasible only if a new theoretical taxonomy is formulated, methodologically rigorous and based on validity checks.

As opposed to the false hope of eXplainable Artificial Intelligence (XAI)^16^, the explanatory power should be based on rigorous validation of digital biomarkers that quantify behavioral decline relevant to brain, body and neurodegenerative social manifestations across the populations. We transform the non-specific symptoms inferred from naturalistic data captured in-the-wild into specific explanations of neurodegeneration-induced behavioral decline. On the one hand, we encapsulate in the definition of the phenotype the symptoms, the dispersion of the symptoms, and a standardized framework for model architecture, validity, reliability and reproducibility. On the other hand, we hypothesize that the definition of phenotypic representations concerning symptom level, temporality, causality and complexity of neurodegenerative diseases will facilitate the translation of the technology from bench to bedside^20^. Apart from early diagnosis, the ultimate hope is to add a dimensional assessment scale alongside the well-defined categories and to search for sensitive and specific biomarkers that aid in rethinking pathogenesis and nosology. Besides that, we aim for an elaboration beyond the correlation between the indexes and the symptoms (clinical states), and rather conclude biological, technical and clinical validation levels. It is the derived latent variables (features) via the analysis of digital biomarkers that can reveal hidden causal factors. In other words, this review fills the gap between empirical research and clinical practice by mapping quantitative traits to a knowledge-supported paradigm.

Moreover, in this review, we attempt to overcome the black-box nature of how AI is currently perceived for refining and organizing our understanding of neurodegeneration manifestations based on behavioral perturbation in-the-wild. This refinement rests on a new standardized framework of multi-level validation for establishing explainable digital phenotyping. First, we report the validity and reliability of digital biomarkers of brain, body and social levels by mapping them to their neurological etiology. The explanations are expanded to include the role of digital biomarkers in defining disease subtypes, accurate definition of thresholds between normal and abnormal and severity estimation. Second, we reinforce the concept of granularity by validating the analysis algorithms and their sensitivity to time and context, decoding inter- and intra-patient variability based on phenotypic and temporal heterogeneity. Third and lastly, we express the importance of reflecting complexity and non-linearity features of neurodegeneration on modeling characteristics and interpreting the interactions between multimodal data for designing decision-making systems with human reasoning power. While a critical emphasis is made on the role of deep learning in neuroimaging, we emphasize the robustness of the deep learning models in analyzing and predicting patient-specific outcomes based on the behavior for the first time^21^. To the best of our knowledge, this is the first review that attempts to establish a theory-grounded taxonomy, one that exploits the power of pattern analysis techniques for early diagnosis and treatment, and that provides three digital biomarkers validation levels, namely: neurobiological, technical and clinical (practical) bases. In this line, we pave the way for the translation of knowledge between empirical research and clinical practice in neurodegenerative diseases, and we rigorously discuss research and clinical opportunities peering into a possible future, with emphasis on experimental design, analysis methodologies, interpretation and data translation to patients in the clinic.

## Methods

### Study design

In this paper, we undertake a “scoping review” strategy whereby we map the literature in the field of neurodegenerative diseases and their behavioral manifestations measured by AI technology in-the-wild to delineate the nature, features and volume of the evolving explainable digital phenotyping field^22^. It should be noted that our intention here is to bring together key concepts from neuroscience, neurodegenerative disease healthcare and AI that, from our perspective, define an emerging taxonomy. In this vein, we attempted to provide qualitative evidence on (1) the theories on behavioral decline in PD and AD, (2) the AI methodologies for measuring and analyzing functional characteristics of neurodegeneration in-the-wild, and (3) the process by which complexity, temporal and phenotyping heterogeneity of neurodegenerative diseases are tackled by AI. With this review design, we aim to reinforce the role of AI validation and explainability for co-created decisions in neurodegenerative disease (PD and AD) healthcare and set up a roadmap for the clinical adoption of digital technology in neurology.

### Review questions

To fulfill the objective of formulating the taxonomy of “explainable digital phenotyping” for neurodegenerative diseases, we attempted to answer three main research questions. To overcome the black-box nature of how AI is currently perceived for personalized neurodegenerative disease characterization, our objectives with this paper are:

1. The definition of the “prodromal behavioral repertoire” based on fine-grained behavioral cues detected in-the-wild and associated with the brain-, body-, and social-level neurodegenerative manifestations.
2. The analysis and validation of computational models in AI that facilitated deciphering the phenotypic and temporal heterogeneity of the neurodegenerative disease.
3. The computational methods of tackling the complex nature of neurodegenerative disease diagnosis, such as uncertainty and heterogeneity from systemic and integrative perspectives.

### Database search strategy

To synthesize the framework of explainable digital phenotyping, we have undertaken a rigorous and replicable search of the relevant literature on AI technology’s role in defining the fine-grained behavioral decline in PD and AD.

**Fig. 2:**
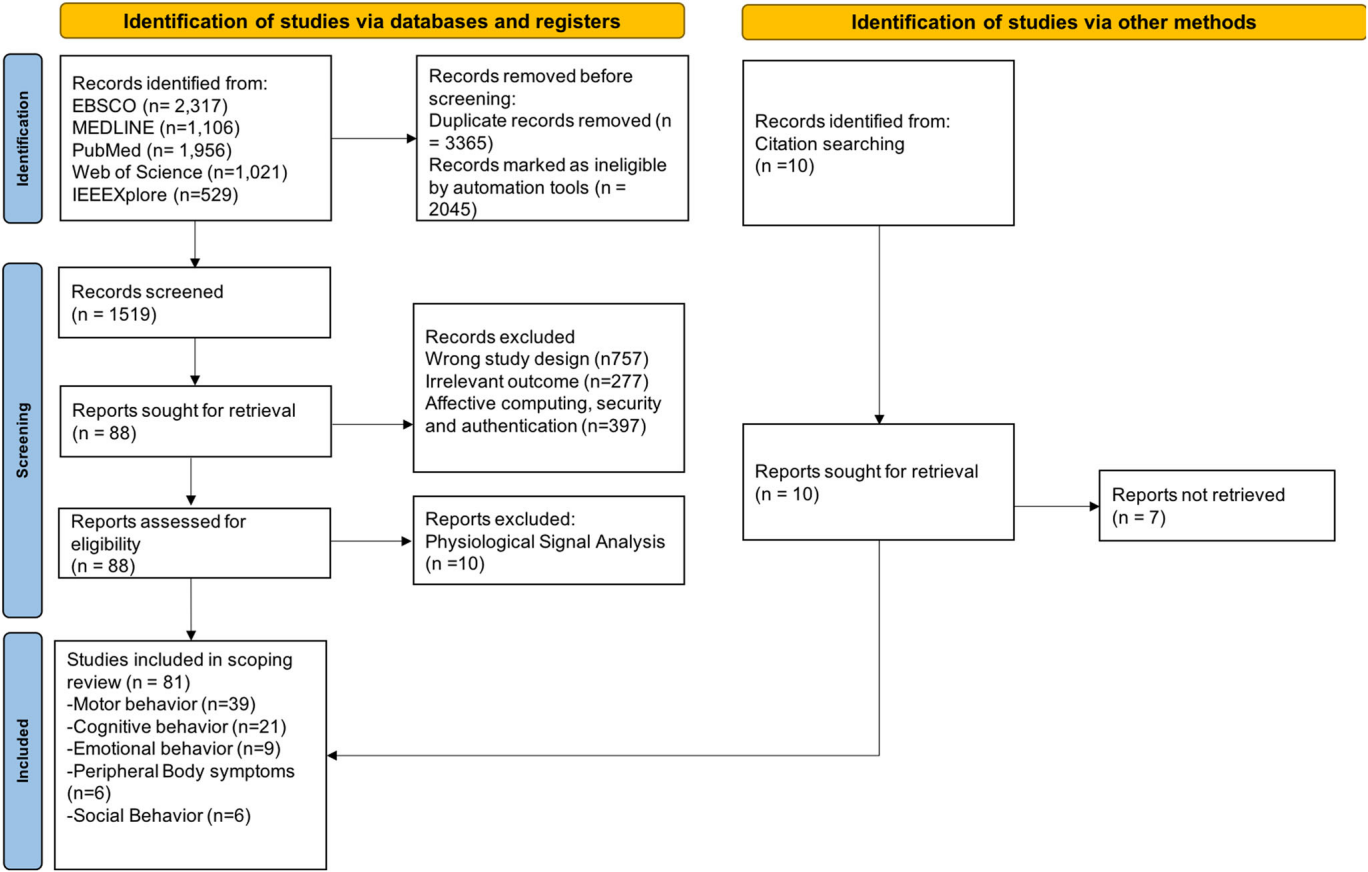
Preferred Reporting Items for Systematic Reviews and Meta-Analysis (PRISMA) 2020 flow diagram for study selection. EBSCO Elton B. Stephens Company, MEDLINE Medical Literature Analysis and Retrieval System Online, PubMed National library of medicine, IEEEXplore Institute of Electrical and Electronics Engineers.

This review follows the Preferred Reporting Items for Systematic Reviews and Meta-Analysis (PRISMA 2020)^23^, and Fig. 2 summarizes the literature search process. Based on the above, we searched for articles published between January 1, 2010, and August 31, 2022, that employed behavioral data collected under clinical or naturalistic settings and used machine learning (ML) algorithms for PD or AD diagnosis and monitoring, without language restrictions.

Searches were conducted via Web of Science, PubMed Medline, and Scopus; the major multidisciplinary databases that report studies on both clinical and engineering domains.

We used the following search terms: [*Neurodegenerative diseases* OR *Parkinson’s disease* OR *Alzheimer’s disease *] AND [*Artificial Intelligence* OR *Machine Learning* OR *Deep Learning*] AND [* Wearable Technology * OR *Home Sensors* OR *Self-Reports*].

The main inclusion criteria are as follows:

1. Peer-reviewed journal articles and conference proceedings.
2. A significant contribution to the digital phenotyping domain for PD or AD, including clear disclosure of the collected behavioral data, valid data processing techniques and statistical and/or AI formulation for symptom monitoring/detection/prediction.
3. Articles utilizing only behavioral data, whether in clinical or ecologically valid settings. Studies employing physiological signals such as ECG, electroencephalogram (EEG) or electrooculographic (EOG) are excluded.

The characteristics of included studies are summarized in Supplementary Table 1.

## Neurodegenerative manifestations

### The brain tier

Neurodegenerative disorders start with progressive loss of neurons due to the accumulation of misfolded proteins in neurons and their surrounding glial cells^24^. Besides the wear and tear changes in the brain with normal ageing, neurodegeneration in the glial cells, for instance, is usually triggered by inflammatory immune responses and over-expression of cytokines in the glial cells decades before the emergence of any symptoms^25^. Furthermore, in the early stages, neuroimaging studies showed that specific brain regions become hyperactive in neurodegenerative diseases, reflecting a compensatory mechanism and perhaps a sign of inefficiency^26^. The synaptic integrity is altered due to neurodegeneration as well, resulting in neural network oscillations with specific spatiotemporal spread pathways, leading to heterogeneous and progressive behavioral change^27^. Disturbances to human behavior constituents, such as emotional, cognitive and sensory-motor regions, are linked to abnormalities in localized brain regions that are anatomically connected. Despite the underlying diagnosis, behavioral disturbances rarely occur in isolation, a mixture of emotional, cognitive, and motor deficits coexist instead^28^. This is attributed to the basal ganglia, a group of subcortical nuclei tightly linked to movement disorders, which are anatomically and functionally subdivided into regions connected to emotional, associative and sensorimotor functions. Consequently, within this integrated network, abnormalities originating from one node result in network-wide perturbations, that reflect on individuals’ behavior and task-specific performance^29^. This concept is also referred to as distributed processing. In addition, a feature that characterizes the basal ganglia nuclei is their high spatial topography, that is, discrete regions of the basal ganglia exert pronounced control on motor and executive functions^30^. More importantly, the segregated loops that form the cortico-basal ganglia-thalamic circuits sub-serve emotional, associative (cognitive) and motor processing. This architecture has been validated in animal studies^30–32^, as well as human studies^33–35^.

Neurodegenerative diseases are accompanied by progressive personality change, referred to as behavioral and psychiatric syndrome^36^. Although previously, the identification of the subtle behavioral changes, such as visuospatial navigation, memory impairment and motor decline, to predict conversion to neurodegeneration has been attempted, the resulting accuracy was deemed clinically low^37^. Stemming from the aim of improved understanding of premorbid behavioral change, in the following, we review the dimensional, fine-grained behavioral disturbances and their neural correlates, derived from remote digital technology, and identify potential assessment and interventional tools based on digital biomarkers, as summarized in Table 1.

**Table 1:** Neurodegenerative manifestations in explainable digital phenotyping based on tier levels.

Tier levels	Neurodegenerative manifestation	Fine-grained behavior	Digital biomarker(s)	Reference(s)
Brain	Limb-kinetic apraxia	Slow and irregular smartphone typing	Keystroke dynamics	^42,54,224^
	Bradykinesia and Tremor (mainly in PD)	Movement slowness and resting tremor	Real-time accelerometer measures	^63,64^
	Speech apraxia	Impairment in speech prosody and phoneme production	Reduced articulation rate, high pauses rate, monopitch and monoloudness	^61,62^
	Gait dysfunction and fall prediction	Freezing of gait, self-induced shifting of body weight, loss of balance	Gait parameters such as velocity, symmetry, intensity and complexity, derived from IMU and accelerometers Step length, step duration and variability	^67–70^
	Aphasia	Poor lexical content and vocabulary size, reduced syntactic complexity, decreased noun rate, high verb and adverb rate	Spontaneous speech tasks Spontaneous written speech, analysis of transcripts, phone calls	^83–85^
	Cognitive impairment	Reduced processing speed, longer response time	Digital biomarkers embedded in serious games	^276^
	Apathy	Hypomimia (mainly in PD) and loss of empathy	Facial expressions through computer vision EMA Voice features	^101–103^
	Idiopathic RBD	Loss of REM sleep muscle atonia Aggressive motor behavior	EEG slowing during wakefulness	^277^
Body	Disrupted circadian activity rhythm	Reduction in executive function (including verbal and categorial fluency). Impaired cognitive function. Sleep–wake rhythm disorder (AD) Reduced circadian activity levels (PD) Motor fluctuation	Actigraphy-derived activity levels	^114,115^
	ON-OFF fluctuations (medication side-effect in PD)	Reduced motion amplitude and irregular activity patterns	Longitudinal recordings from noninvasive motor sensors	^72^
	Reduced sleep quality	Difficulty falling asleep and early awakening Sleep duration	EEG spindle EEG complexity	^117,119,121^.
Social	Cognitive ToM	Inability to infer and respond to others’ cognitive behavior	Engagement in enriched virtual environments	^215,278^
	Affective ToM	loss of empathy, inability to infer and respond to others’ emotions	Social communication frequency	^279^

*PD* Parkinson’s disease, *EMA* ecological momentary assessment, *ToM* Theory of Mind, *REM* rapid eye movement, *RBD* REM behavioral sleep disorder.

#### Motor

Neurodegenerative diseases are characterized by progressive motor decline due to oxidative stress, cellular damage^38^, and the depletion of the dopaminergic neurons in the substantia nigra (SN) pars compacta^39^. These symptoms are usually mild at early stages, therefore, detected at advanced stages after progressing to gross motor symptoms. A natural consequence of this is to look for surrogate motor symptoms, derived from naturalistic behavioral characteristics, that affect fine dexterity skills. Dexterity loss and fine motor impairment, also referred to as limb-kinetic-apraxia^40^, constitute a central feature of cortico-basal disorders, particularly those combining parkinsonism and cognitive decline^41^. Besides that, compelling evidence suggests that this feature carries different representations depending on the extent of pathological spread, and is a marker of disease progression. While these definitions date back to the 1920s, there is currently no standardized clinical assessment to detect such deficits, especially in the elderly population. To this end, we review the clinically relevant motor variables derived from passively acquired smartphone typing parameters, known as keystroke dynamics^42–44^. The search for high throughput, naturalistic tasks for motor behavior analysis came about not only to increase the participant pool, but also to minimize the Hawthorne effect. The proliferation of smartphones and the growing interest in embracing technology for healthcare allowed the exploitation of passively collected data. To this end, human-device interaction patterns, when pulled together via statistical and ML models, uncover comprehensive, personalized behavioral profiles^45^. Cross-sectional and longitudinal studies reveal disease-related abnormal behaviors, and deviation from baseline. In particular, the assessment of fine motor skills is realized by finger dexterity upon typing, which became a real-life task. It has been well agreed upon that consistency of movements and the influence of timing are highly tied to the motor cortex and thalamic attention. It is for this reason that the typing behavior is hypothesized to embed symptom-specific indexes, in line with standardized medical scales, and finger dexterity tests. In any case, the ultimate objective of these models is the identification of early warning signs, that correspond to disease-induced transitions in behavior^46^. The realization of this goal starts with designing behavioral experiments that facilitate “zooming-in” to extract behavioral features, with plausible connections to disease symptoms and clinical scales. In this realm, passively collected typing kinetics offered a rich set of clinically meaningful and practically useful variables, analogous to those examined during finger dexterity tests^47^. Keystroke dynamics have been employed as a promising modality for neurodegenerative disease feature extraction^48^. The hallmark motor symptoms of PD have therefore been at the center of this paradigm, stemming from the hypothesis that implies the impact of early disease signs on fine motor control and dexterity. The pioneering work is that developed by Giancardo et al., whereby the statistical features derived from the Hold Time (HT) are used to create an automated diagnosis metric via an ensemble regression model, the neuroQWERTY, that did not only discriminate early PD patients from healthy controls but also de novo PD patients^49^. Early studies in the field particularly targeted HT due to its insensitivity to typing skills, but as this area gained attention, the search for new features continued. Second and higher (>2)-order statistics (covariance, skewness, kurtosis) derived from the normalized Flight Time (nFT), which is the latency between releasing a key and pressing the next one, enhanced the interpretability of the PD features^50^. Besides slow movement and temporal dispersion, PD patients are characterized by heteroscedasticity, that is, inconsistent variation (in the FT) along the time. Taking the analysis forward, multivariate ML models that combine HT, nFT and the normalized Pressure (nP) applied to the keys while typing have been validated in^51^. Whereas the aforementioned studies collected and validated the diagnostic methods in-the-clinic, the transferability of the models to data collected in-the-wild has also been validated^42,52,53^. This was the first step toward the validation of unsupervised diagnosis of PD in uncontrolled environments, in which sampling and confounding factors induce inevitable noise. Another breakthrough in the area was the adoption of multimodal analysis. Data obtained from touchscreen typing and accelerometer data were unobtrusively collected from early-stage PD patients to estimate fine motor impairment and tremor symptoms, respectively^54^. Besides that, mild cognitive impairment (MCI), considered a precursor to dementia and AD, was also targeted by multimodal analysis. MCI is characterized by a decline in memory, lexical processing and motor decline, but remains hardly distinguished from normal ageing using current clinical diagnosis scales^55^. With the hope of improving early detection of MCI symptoms, multimodal analysis of motor and linguistic features offered promising results^56,57^. Recently, typing activity was also validated to discriminate the severity level of PD using unsupervised clustering based on the UPDRS-III and the Patient Health Questionnaire (PHQ-8)^58^.

Furthermore, neurodegenerative diseases are characterized by insidious apraxia of speech, that worsens with time^59^. Apraxia is mainly associated with abnormal motor planning, and processing of speech, independent of linguistic features, resulting in phonetically and prosodically impaired speech. Speech apraxia can sometimes be the only manifestation of neurodegeneration, even in the absence of obvious movement disorder signs, and has been linked to tau pathology and the white matter followed by gray matter degeneration. This implies that early apraxia manifestations progressively change to aphasia, involving linguistic domains. For instance, in idiopathic REM sleep behavior disorder (iRBD), speech impairment constitutes the first Parkinsonism signs^60^, motivating the development of digital biomarkers deciphering acoustic features indicative of apraxia^61^. For example, employing acoustic features from multilingual populations conveyed their language-independent longitudinal, discriminatory performance for iRBD and PD. Hypokinetic dysarthria, for instance, was evaluated based on the harmonics-to-noise ratio (harsh noise), intensity and pitch variability (monoloudness and monopitch), articulation rate and pauses. Most recently, PD diagnosis was passively undertaken based on phone calls via a language-aware ML framework^62^. Given that speech apraxia is independent of language, the accuracy of digital biomarkers was validated across centers with different European languages and no differences were found between them.

Accelerometer data proved feasible in quantifying the rigidity, bradykinesia and tremor in PD^63^. For example, recordings from six sensors for the upper extremity, while performing naturalistic home activities, harnessed by an ensemble of random forests and convolutional neural networks, returned a high detection rate of tremor and bradykinesia^64^. In community-dwelling elderly, falls resemble a frequent cause of physical injury and influence mental health. Subsequently, on the basis of inertial measurement unit data, logistic model tree predicted fall prediction risk by employing meaningful features describing mobility and frequency of daily activities^65^. Besides that, using video analysis through home-installed digital cameras, fall features were extracted, analyzed and labeled by communicating with caregivers at the incidence of a fall. These include self-induced shifting of body weight, failure to achieve a balance and freezing of gait. These characteristics and longitudinal remote fall monitoring convey the validity of remote gait assessment for fall prediction^66^. Remote monitoring of mobility and fall prediction models have been formulated^67,68^. Specifically, the impact of neurodegeneration on the spatiotemporal dynamics of gait, including pace, rhythm, postural control, balance and step-to-step variability, entails not only multifaceted quantification and analysis systems, but also deciphering longitudinal and inter-patient variability, for subsequent fall prediction and rehabilitation paradigms. To convey the inter-group variability in step length, step duration and variability, analysis of variance (ANOVA) was employed to separate healthy controls from PD patients^69^. Taking the analysis a leap forward, the detection of gait freezing episodes was performed by deep learning based on accelerometer data revealing the potentiality of such methods in defining risk assessment frameworks that are otherwise unfeasible^70^.

Motor activity monitoring has also been used for the titration of medication. In PD, it is well known that patients exhibit on-off states characterized by dyskinesia and motor fluctuation that differs depending on the limb^71^. Subsequently, a dataset for limb-specific medication response sensitivity has been recently formulated based on the fusion of data collected in-the-clinic and data collected in-the-wild including 4-day accelerometer data collected from the trunk, forearms and lower back^72^.

#### Cognition

Cognitive inertia refers to impairments in planning, working memory, rule-finding and set-shifting, which, in turn, result in apathetic behavior. The leading cause of cognitive decline is AD and Lewy body neurodegenerative disorders^73^. In particular, MCI constitutes an intermediate stage between normal and pathological ageing, therefore, remains hardly detected but is usually reported by caregivers without specific clinical biomarkers. Furthermore, in PD, cognitive decline is among the major non-motor symptoms with a profound impact on quality of life^74^ attributed to the spread of Lewy body even before the onset of motor symptoms^75^. For early detection of cognitive decline, a computerized battery of tests that estimate cognitive stability indexes targeting memory, attention and response time domains have been designed^76^. Subsequently, embedding cognitive impairment evaluation in virtual game environments not only assists in assessing cognitive function, but also aids learning and motivational aspects. For example, in Klondike Solitaire, the player actions relative to game characteristics (i.e., movements, game outcome, difficulty level) are transformed into multifaceted digital biomarkers assessing cognitive function related to processing time, performance, error, and executive function^77^. Furthermore, the merger of virtual reality (VR) and AI facilitated the continuous assessment of instrumental activities of daily life, by analyzing the kinematic patterns of the head and the hand during real-life tasks, resulting in the identification of behavioral measures capable of predicting MCI, yet not necessarily correlated with conventional neurological questionnaires^78^. Away from this dichotomous view, the assessment of MCI should ideally be continuous and multimodal, revealing subtle neural perturbation^79^. Within the cognitive domain, deficits in linguistic abnormalities, such as progressive aphasia, are usually present in neurodegenerative disorders^80^. Speech analysis through deep neural networks proved successful in the binary classification of AD^81^. Computing rhythmic, acoustic, lexical, morpho-syntactic, and syntactic features of spontaneous speech transcribed via natural language processing (NLP) discriminated early MCI, multi-domain MCI from healthy controls, not only indicating the classification of subtypes, but also the sensitivity of the method to disease progression^82^. NLP models allowed multimodal analysis of fused speech and text-linguistic features for enhanced discriminatory performance as in transformer-based. Besides that, ensemble models for training acoustic and linguistic features used majority voting resulting in a discriminatory performance of 81%^83^. Furthermore, the detection of early dementia symptoms and the prediction of the mini-mental state examination (MMSE) based on speech and transcripts data were achieved with an accuracy of 90% and a root mean squared error of 3.61, employing recent trends in multimodal deep learning of textual and speech data^84^. As opposed to the use of majority voting or probability averaging to make a final prediction^85^, an end-to-end deep learning model was developed comprising bidirectional encoder representation transformer and vision-based transformer, co-attention, multimodal shifting gate and self-attention mechanism to learn the interactions between textual and speech features^84^. Cognitive impairment usually accompanies PD motor symptoms as well; therefore, real-life context influence on PD patients’ cognition was investigated. Using digitized ecological momentary assessment (EMA) with a smartphone application for assessing working memory and executive function of PD patients at-home, cognitive function was evaluated along with the impact of neuropsychological symptoms^86^. Interestingly, PD patients showed high working memory and executive function performance, as inferred by the Backwards Spatial Spans task and the Trials-B task within the application. Furthermore, the smartphone application allowed the analysis of these behavioral domains with varied locations, time-of-the-day and social context, constituting an avenue for future inter-patient and intra-patient variability analysis.

Taking the analysis of cognitive function a leap forward, the impact of circadian rhythm has been explored via mixed-effect models in patients with AD^87^. This was achieved by frequent ambulatory assessment (self-reported activity) over 7 days. The impact of diurnal patterns in those with cerebrospinal fluid biomarkers was also investigated and showed that they performed worse during evening hours. In accordance with previous studies^88^, the results showed that patients with AD exhibit a reduction in memory performance and response inhibition late during the day, but not in spatial navigation or processing speed. Furthermore, compared to studies that utilized conventional cognitive assessment and failed to correlate cognitive function to daily fatigue^89,90^, there was a weak correlation between subjective feelings of fatigue and cognitive performance. Conversely, no relationship was identified between mood and cognitive performance.

Besides cognitive assessment, a randomized control trial (RCT) was conducted to assess improved cognitive function in the elderly after 12 months of cognitive training via Application-based Cognitive Training at Home^91^. Daily smartphone-based training for 30 min improved memory, attention, language, visuospatial and executive function in the intervention group over the year. It is also well known that neurodegenerative diseases are characterized by a decline in speech production, also known as aphasia^92^. Spontaneous speech tasks have been adopted to detect aphasia, whereby patients are asked open-ended questions such as telling a story or describing an image^93^. A battery of linguistic skills quantification is performed and evaluated subsequently. Given the impact of ageing on dynamic balance control, the quantity and quality of mobility have been employed to detect cognitive decline in patients with AD^94^. Compared to gait analysis paradigms for locomotion analysis, the quality of turning during daily activity proved to be a reliable predictor of cognitive impairment. This is in line with the previously reported relationship between sensor-based walking sensors and attention, processing speed and global cognition^95^.

#### Emotion

Emotions are transient states that synchronize with changes in physiological arousal, motor expression, subjective feeling, action tendencies and cognitive processes, as a result of external (environmental) or internal stimuli^96^. Deficits in the reward processing neurocircuitry have always been linked to negative emotions, that is, the responses to what should be positive stimuli. Alterations in the amino-acid neurotransmitter, glutamate aminobutyric acid, which plays a key role in regulating dopaminergic pathways within the ventral tegmental area, are usually altered in patients with dysregulated emotions^97^. Moreover, dysfunction of the prefrontal-basal ganglia systems results in affective disturbances that disorganize behavior, virtually in all neurological disorders^98^. Neurodegenerative diseases, in particular, are characterized by apathy, a symptom with debilitating consequences on the quality of life, linked to a reduction in goal-directed behavior, loss of interest and initiation of activities in addition to emotional blunting. Early identification and treatment of apathy results in improved quality of life and executive function, and reduced caregiver burden^99^. Although a triad of methods and scales for measuring apathy are proposed, there needs to be more certainty in their validity and reliability^100^. Tremendous effort has been made to understand and target apathy for pharmacological and non-pharmacological interventions. Yet, more emphasis should be devoted to quantifying its biomarkers, including more precise descriptions of its ecological manifestations. To this end, we assess the reliability of digital biomarkers for detecting apathy in-the-wild. Apathy has been remotely evaluated by EMA^101^, voice features^102^ and most recently by facial expressions^103^. Specifically, the advances in computer vision methods allowed the investigation of apathy severity given by the Apathy Inventory scale through the analysis of the intensity and frequency of Action Units extracted from videotapes of the elderly recalling positive and negative memories, which was extended to assess hypomimia in PD^104^ and lack of empathy in AD^105^. Given the prevalence of depression in PD^106^, it was predicted using a simple support vector machine model that employed the national Korean Biobank registry of PD patients along with their health habits, socio-demographics, neuropsychiatric and sleep disorder symptoms^107^.

The use of actigraphy that quantifies the three-dimensional movement of the arm has also been harnessed for detecting apathy in-the-wild. Over a 7-day analysis of the movement activity of AD patients, several parameters, including daytime mean motor activity, daytime napping, and nighttime motor activity, were extracted from the actigraphy and analyzed by post hoc ANOVA^108^. The results indicated that apathy is associated with low daytime motor activity^109^ and shorter durations of daytime napping. On the contrary, Mulin et al.^110^ found a proportional relationship between high durations of daytime sleepiness and reduced goal-directed cognitive activity and apathy that, in turn, increases dementia severity. These mixed interpretations call for a more rigorous analysis of motor behavior and its association with apathy.

### The body tier

Real-time detection of changes in vital signs reflects early disease symptoms attributed to neurodegeneration^111^. Neurodegenerative manifestations stem from autonomic and peripheral systems, and their digital representations are presented in this section. While most wrist-worn devices are employed in analyzing motor function, their analysis has been extended to predict non-motor symptoms. For instance, multiple linear regression models were performed in a retrospective study^112^ to correlate bradykinesia and dyskinesia detected from the PD Kinetigraph with non-motor symptoms. The results indicated that bradykinesia was significantly associated with constipation and sexual impairment, while dyskinesia predicts cognition and mood. The noninvasive monitoring tools extend to incorporate organic graphene sensors and micro-fluidic systems capable of monitoring stress levels, for instance, thereby amalgamating the electro-chemical sensing of sweat with mobile health (mHealth)^113^. This implication suggests a promising avenue for remote analysis of diurnal patterns and responses to stress. Neurodegenerative manifestations also include impaired circadian rhythm due to the degeneration of the supranuclear thalamus^114^. In particular, weak circadian activity levels are linked to the predisposition of amyloid beta- and alpha-synuclein, thereby correlated with specific behavioral markers. Actigraphy-derived measurements as evaluators of circadian activity, such as the amplitude of motion, mesor, and motion rhythmicity, were used as predictors of cognitive decline over 5 years. The latter was assessed by the MMSE, California Verbal Learning Task, digit span, categorial and verbal fluency. It has been concluded that disruption in circadian activity, measured by actigraphy, is linked to poor cognitive performance, especially in the executive function domain^115^.

Circadian rhythm manifestation should always be distinguished from those of sleep patterns. To reflect the sleep fragmentation characteristic of neurodegeneration^116^, sleep staging from EEG has been used to convey the lower non-REM stability and high REM/non-REM transition rate in PD^117^. Some studies pointed out the beneficial effect of short daytime napping (<30 min) on cognitive function^118^. Reduced sleep quality has been linked to cognitive decline^119^. As a result, sleep onset latency, total sleep time, and apnea-hypopnea index were derived via NLP of 61,165 free-text polysomnography (PSG) reports from the Houston national electronic health records (EHRs) from 2000 to 2019 to discriminate patients at risk of dementia^120^. In electrophysiological measures, reduced sleep spindles quantified from EEG analysis of stage-2 sleep activity have also been identified as robust markers for cognitive decline in neurodegenerative diseases^121^. Besides PSG, movement analysis during sleep has also been used to evaluate sleep quality. However, it is unlikely that the multifaceted behavioral and physiological sensors during sleep be easily exploited, given the burden on patients^122^. Remote home sensors could be used instead. Autonomic dysfunction has been a famous measurement target in neurodegenerative diseases, such as heart rate variability^123^ and respiratory signal analysis^124^.

Disruption in circadian rhythm and sleep quality has also been linked to another symptom in neurodegenerative diseases: chronic pain^125^. It has also been concluded that the prevalence of pain in neurodegenerative disease patients is linked to a decline in cognitive performance, psychomotor behavior and executive function^126^. For example, in patients with AD, pain processing has been shown to increase^127^. To prove this, imaging biomarkers and facial expression features (extracted from the Facial Action Coding System), in addition to self-reports to label pain levels, have been co-processed^128^. The results revealed that cognitively impaired patients with high levels of pain are characterized by reduced gray matter volume in the medial orbitofrontal and the anterior cingulate cortex.

### The social tier

Social inappropriateness is frequently the first manifestation of neurodegeneration, especially in AD^129^. According to the Theory of Mind (ToM) and its neural basis, neurodegenerative disease induces different patterns of affective and cognitive ToM deficits^130^, reflecting on the social behavior of neurodegenerative disease patients^131^. In particular, social cognition, including self-knowledge, perception of others’ emotions and beliefs, communication and interpersonal motivations, are also impacted by neurodegenerative diseases, which adversely affect social life. Despite this, understanding the association between cognition and social skills and how these patterns differ between disorders and individuals remains a challenge^132^. In the context of social cognitive manifestations in neurodegenerative diseases, we can define six symptoms as follows: (1) early behavioral disinhibition and inappropriateness, (2) apathy, (3) decline in empathy, (4) compulsive behavior (i.e., repetitive movements and stereotypy speech), (5) hyper-orality and dietary changes, and lastly (6) executive dysfunction^133^.

The social cognitive performance of AD patients was evaluated based on the Ekman 60 faces test and story-based empathy test. The former implies the selection of emotional labels for face photos of ten actors with six basic emotions. During the latter, the selection of the correct story ending is required^134^. Compared to healthy controls, AD patients had lower performance in all tasks, but more importantly, they reported higher discrimination inferred by the affective tasks compared to those assessing cognitive attribution. This implies the importance of evaluating surrogate social cognition markers of neurodegeneration, as no reliable cognitive ToM tasks exist. For this purpose, the passive analysis of smartphone data, such as the frequency of phone calls and mobile applications, facilitated the identification of patients with cognitive impairment from age- and education-matched healthy individuals thus far^135^. For instance, those with cognitive impairment had less usage activity and contacted the same people more frequently.

Targeting the social interactions domain of neurodegenerative disease populations, the concept of “Enriched Environments” achieved by video games can reinforce social engagement, and preserve cognitive function^136^. Specifically, environmental enrichment refers to home conditions that facilitate sensory, motor and cognitive performance and encapsulate the enhancement of social interactions within a unitary framework^137^. In particular, the latest gamification platforms enhance social interactions and entertainment besides improving adherence to rehabilitation^138^. Such a game-buddy system, which involves two or more patients with the same age, diagnosis, and disease stage, is hypothesized to reinforce social and community feeling while providing a positive environment for physical rehabilitation. Another advancement in this realm lies in the development of Artificial Robots capable of perceiving and reacting to human emotions, thereby providing an assistive architecture for maintaining cognitive and emotional intelligence during social interactions^139^. Future work is still needed to investigate the impact of social learning^140^, delivered by digital technology, on the social behavior of neurodegenerative disease patients.

While gamification and VR have been proposed as potential cognitive rehabilitation strategies for neurodegenerative diseases, clinical trials did not show their therapeutic value yet thus far. Among the innovative therapeutics for neurodegenerative diseases that have emerged lately is noninvasive brain stimulation (NIBS) approaches^141^. Ideally, such therapeutics should function at the degeneration level as NIBS enables brain plasticity to modulate cognitive processes. Moreover, NIBS approaches should be applied early in the disease course to yield an optimal outcome and treat patients before the degeneration of substantial neural tissue. Currently, there are two commonly employed NIBS approaches transcranial magnetic stimulation (TMS) and transcranial direct current stimulation (tDCS). Developed by Anthony Barker in 1985, TMS employs a brief-lasting magnetic field to probe correlation links between cortical areas, by stimulating action potentials in specific cortical regions^142^. In contrast, tDCS is based on passing a direct current between two electrodes on the skull, resulting in polarized target brain regions, eventually leading to altered resting membrane potentials. Both approaches have been used for a multitude of neurodegenerative diseases. For instance, in AD, tDCS in the temporal-parietal lobe proved effective in reversing memory processing deficits by analyzing the induced changes in the spectral characteristics of EEG before and after therapy^143^. These results boosted the popularity of tDCS in home environments for the sustained cognitive performance of early AD patients^144^. Consistent with the pathophysiological characteristics of brain dysconnectivity in prodromal AD, repetitive TMS results in selective modification of cognitive functions such as episodic memory. Besides neuropsychiatric behavioral analysis, the positive outcome of neurostimulation has also been confirmed by significant correlations between tau protein levels such as amyloid beta and cognitive imporovement^145^. Overall, these findings confirm the feasibility of noninvasive neurostimulation in mitigating network dysfunction in AD. Similarly, in PD, TMS targeting the prefrontal cortex results in adaptive motor function improvement and mood swings^146^. Moreover, repeated tDCS over the dorsolateral prefrontal cortex, when combined with physical therapy, improves the long-term physical and cognitive performance for up to 3 months of PD patients with MCI^147^. Such preliminary evidence holds promise for designing holistic rehabilitation programs in early neurodegenerative disease, but further work on formulating personalized behavioral change protocols is still required. In recent years, information-based neurostimulation emerged as a refinement of understanding how brain stimulation alters connectivity and functions in neurodegenerative diseases^148^. This notion proves essential for enhancing the therapeutic outcome of noninvasive brain stimulation by tailoring brain activity patterns and NIBS parameters at the time of stimulation. To increase neurostimulation outcome specificity, detailed, task-evoked brain activity patterns that encode cognitive, emotional and motor functions over the disease course should be monitored and modulated^149^. Regarding the control of NIBS parameters, frequency-tuned interventions have shown special clinical interest. For example, Parkinsonian tremor is mitigated when tDCS is applied to the motor cortex at tremor frequency^150^. Novel NIBS therapeutics for neurodegenerative diseases continue to evolve in the clinical and home environment. Still, the targeted populations are pathologically too far advanced in the disease course, precluding robust neuroprotective impact. Hence, deciphering early neurodegenerative manifestations, as advocated in this review, is likely to identify patients long before the substantial neural loss. Notably, PD patients with intractable motor fluctuations and who experience adverse medication events are considered good candidates for deep brain stimulation (DBS)^151^. DBS is a minimally invasive surgical treatment whereby the subthalamic nuclei are targeted to be the location of electrode placement. DBS results in long-lasting improvements in tremors and dyskinesia.

## Granularity and continuity

While it is well known that neurodegenerative diseases are characterized by pronounced heterogeneity that impedes accurate stratification and prognostication, we hereby discuss the role of digital technology in decoding inter- and intra-patient variability. To construct a comprehensive picture of this heterogeneity and the resulting biomarkers variance, we review the AI-based methodological approaches to separate phenotypic from temporal heterogeneity based on cross-sectional and longitudinal behavioral analysis, respectively^152^. In this way, we provide meaningful insights into the neurodegenerative mechanisms that take place in-the-wild by applying optimal granularity parameters to naturalistic behavioral data. Moreover, in the context of fine-grained explanations, detecting spatiotemporal events constitutes another venue^153^. In this vein, although the concept of continuous monitoring is claimed to be achieved, the complete definition of spatiotemporal events, symptom sensitivity to real-life contexts and other environmental stressors still need to be discovered. For this purpose, we include in this section the characteristics of spatial, temporal, and environmental and context-related information and their role in resembling intelligent, cause and consequence systems. We show that once we approach the rich data streams with a comprehensive understanding of data quality, analysis windows, causality between variables and algorithm characteristics, we can transform the non-specific traits into more specific syndromes. For the first time, this is achieved by defining phenotypic and temporal heterogeneity of neurodegenerative disorders on the basis of remote behavioral data.

### Phenotypic heterogeneity

The degeneracy of the human brain^154^ and the complex interaction between the genetic background and environmental factors result in tremendous variability in the symptom characteristics. Instead of standard, unsupervised clustering methods that separate heterogeneous patient groups along the axis of highest variability that might not necessarily be related to disease characteristics^155^, we focus on unbiased data-driven approaches in defining disease subtypes based on specific features derived from naturalistic data. Given the crosstalk in speech impairment that occurs in neurodegenerative disorders, disease-specific acoustic features of hypokinetic, ataxic and spastic speech impairment characteristics were used to classify Multiple System Atrophy parkinsonian and cerebellar subtypes (MSA-P, MSA-C) from PD^156^. Furthermore, to account for PD progression during patient subtyping and away from the conventional clustering algorithms, multidimensional longitudinal characteristics from the Parkinson Progression Initiative Marker data are incorporated in a long-short-term memory followed by dynamic time warping framework, thereby providing clinically relevant temporal trends of patients in each class. The PD patients were subsequently classified into mild, moderate and severe progression regardless of the symptom severity at baseline, reflecting that disease severity and progression rate are independent disease characteristics^157^. Given the evidence of broader degeneration and more severe symptoms in PD patients with rapid eye movement sleep behavior disorder, defining PD subtypes on this basis was accomplished by an ensemble random forests model of EEG and EOG^158^. Specifically, a multidimensional data-driven approach of ensemble random forests was employed to track the prognosis of de novo PD patients and detect RBD in PD^158^. The system performed automatic micro- (5 s epochs) and macro-sleep (30 s epochs) staging and extracted EEG spectral and complexity features, EOG energy features given its relevance to eye movement energy and micro-sleep (e.g., transition and stability indexes) features. The classification results indicated the influence of RBD on the EEG and EOG patterns among PD patients and that the best-performing features are those extracted from the micro-sleep staging, particularly the wake-sleep transition index. Future clinical trials are still needed, however, to determine if idiopathic RBD is a subtype or a prodromal phase of PD. Another condition with significant overlap with PD is Essential Tremor (ET), given that the rate of misdiagnosis of the two conditions is up to 30%^159^. ET was distinguished from PD based on the complexity features of EMG signals obtained from the biceps brachii muscles and the kinematics of arms^160^.

Another area for improvement of the current screening tools is their inability to identify subjects with suboptimal treatment responses in real-life settings. To fulfill this aim, a retrospective study gathered data from the Adelphi Parkinson’s Disease-Specific Program (DSP) in seven countries (i.e., France, Germany, Italy, Japan, Spain, UK and US) to detect PD patients who satisfy the 5-2-1 criteria^161^. The latter describes PD patients who take more than five doses of levodopa daily, experience longer than 2 h OFF state and more than 1 h of debilitating dyskinesia. The data included the clinical assessments and outcomes reported by the physician (clinical burden) and the self-reported information of the patients and their caregivers, including demographics, treatment condition and quality of life (humanistic burden). The results indicated that 5-2-1 positive PD patients had higher clinical and humanistic burdens. With the same goal, the MANAGE-PD system driven by data from PD experts panel to identify patients with suboptimal symptoms controls^162^. This type of analysis is particularly essential because as the disease progresses, the need for individualized assessment and treatment is higher^163^. Moreover, the longitudinal comparison of gait parameters to baseline uncovered per-subtype progression trends, with a more significant gait decline in the TD compared to the PIGD phenotype. Nevertheless, such subtypes reflect only a limited view of the true symptoms’ heterogeneity of PD in real-life practice according to a wide range of related symptoms. These models also lack internal heterogeneity and are based on a single entity for the separation of PD subtypes, Consequently, multi-partitioning clustering was used to decipher PD subtypes and analyze the associations between them using data collected from the Movement Disorder Society Non-Motor Rating Scale (MDS-NMS) comprising eight partitions^164^. The model leverages conditional linear Gaussian Bayesian Networks (GBN) that learn latent variables of every partition. The reason behind using Bayesian networks includes the graphical representation that allows interpretations of the relationship between variables and their partitions and the conditional dependencies between partitions. Eventually, eight partitions of motor and non-motor symptoms were defined; impulse control issues, non-motor symptoms including apathy, gastrointestinal, sleep and urinary dysfunction, dyskinesias and psychosis, mental and physical fatigue, axial symptoms, loss of smell and motor fluctuations, autonomic dysfunction, depression and loss weight, and finally anxiety with excessive sweating.

Besides PD subtypes classification, the cognitive profile differences between MCI due to Lewy bodies (MCI-LB) and that progressing to dementia (MCI-AD) have been disentangled via longitudinal assessment of clinical characteristics and executive function^165^. Employing linear mixed models revealed the distinct profile of sharper decline in executive function and attention in MCI-LB compared to MCI-AD with memory decline. Yet, both had similar progression rates and times. We report the sparse studies devoted to MCI, dementia and AD heterogeneity analysis, thereby stressing the need for a more rigorous analysis of the heterogeneous cognitive phenotyping.

### Temporal heterogeneity

In light of the dynamics system conceptualization^166,167^, wherein emotional, cognitive, and motor (behavioral) concomitants interact, forming processes that change over time, establishing the clinical phenomenology via temporal pattern analysis became essential. However, the latter is a matter of empirical research and a translation of knowledge toward clinical practice still needs to be clarified. The formulation of patient-specific case conceptualization, is the route to patient-optimized intervention targets for neuromodulation and neuroprotection^168^.

**Fig. 3:**
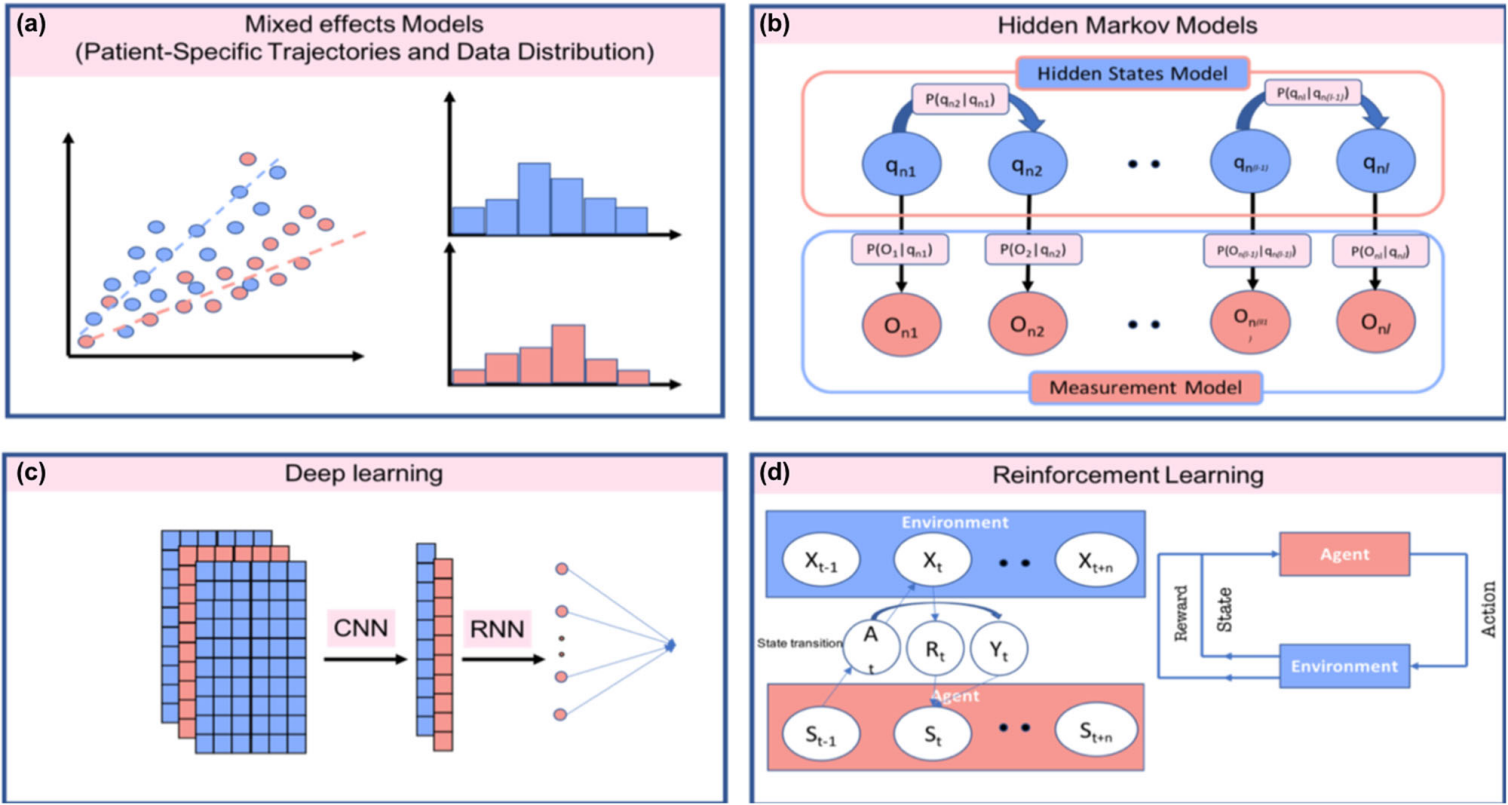
Computational models for dynamic behavioral analysis and disease progression models. **a** Linear mixed-effect models are one of the first models employed to learn patient-specific data distribution and patterns. **b** Hidden Markov Models are statistical models for sequences of data that learn hidden states (*q*) given present observations (*O*). **c** Deep neural networks such as convolutional (CNN) and recurrent neural networks (RNN) learn personalized and temporal data patterns, respectively. **d** Reinforcement learning is an optimization model based on a reward function (*R*) relating environmental series (*X*) with predicted states (*S*) through an action (*A*), such that the cumulative reward is maximized across time (*t*).

Longitudinal data analysis aims to learn the sequence and the ordering of the time-dependent patient characteristics concerning disease progression. This implies the shift to hypothetical models of dynamic biomarkers^169^ as an ambitious avenue yet with significant methodological challenges, especially since the progression of the symptoms is nonlinear^170^, and that until now, the definition of “early” and “long” disease duration is elusive. Owing to the definition of patient-specific trajectories, it should be noted that the collected biomarker data belongs to a high dimensional space, and to a specific time point of the trajectory. To this end, disentangling spatiotemporal patterns in longitudinal data has been achieved by longitudinal deep learning models that do not depend on users’ age as the case in mixed-effects models and are based solely on the ordering of the phenotypic information^171^. This was achieved via an architecture pivoted on a self-supervised neural network model (see Fig. 3).

Understanding the evolution of the symptoms at early stages is particularly complex compared to moderate to advanced stages. Worth mentioning here is the progression of idiopathic RBD to alpha-synucleinopathy, such as PD and dementia with Lewy Bodies (DBL). Accordingly, the prognosis of neurodegeneration in RBD patients was determined based on decreasing algorithmic complexity of the EEG^172^. Besides that, adopting recurrent neural networks for temporal pattern analysis and EEG classification yielded high discriminatory potential^173^. In particular, to process the dynamic features of EEG, the Echo State Network employs nonlinear dynamics. It processes the temporal features of the EEG across multi-channel spectral (frequency) inputs with random but fixed network weights to enhance training efficiency. To mitigate the computational cost of such “dynamic reservoir neural networks”, a deep convolutional neural network was designed for multi-channel EEG analysis and to localize parts of the EEG input that excite the network nodes the most^174^. Detection of REM sleep behavior events on a longitudinal basis also supports the increasing severity of the symptoms^175,176^, supporting the mono-exponential degeneration of the SN during prodromal PD^177^. This approach is yet to be replicated on behavioral data obtained in-the-wild.

Furthermore, in a group of early incident PD, a battery of gait characteristics was used to assess impairment over 18 months for postural instability and gait difficulty (PIGD) and tremor-dominant (TD) subtypes, and the influence of Levodopa equivalent daily dose^178^. The fact that some gait characteristics, such as pace, swing time and posture control, were refractory to levodopa confirms the non-dopaminergic pathology behind these domains in PD. It suggests the need for surrogate treatments, beyond those targeting motor behavior, focusing on cognitive domains. In addition to symptom detection and monitoring, multivariate mixed-effects models were utilized to assess long-term exercise’s impact and physical training’s intensity on PD clinical course^179^. Using the Parkinson’s Progression Markers Initiative data of 237 early-stage patients, class-II evidence on the association of long-term physical activity high levels with slower PD progression rate over a 6-year follow-up, especially when it comes to postural instability and gait quality. In accordance with the previously reported fact that different types of exercise are linked to different neuroplasticity mechanisms^180^, Tsukita et al.^179^ concluded that exercise habits that require sophisticated balance, such as dancing and aerobics, are associated with the slowest symptom deterioration.

The Oregon Center of Aging and Technology (ORCATECH) (https://www.ohsu.edu/oregon-center-for-aging-and-technology), a home-based innovative research endeavor to collect real-life continuous data analysis for healthy ageing, is an example that facilitated longitudinal monitoring^181^. Employing high-frequency patient-specific distributions of executive function outcomes in home settings not only distinguished MCI trajectories to AD from healthy controls but also proved that high-frequency data acquisition offsets the need for a large sample size, which is a grand challenge in data mining^182^. The data included weekly mean walking speed, walking speed variability (obtained via in-home passive infrared motion sensors) and time spent on computer usage from 119 cognitively healthy old adults followed for over 3 years to identify conversion to MCI. Using patient-specific thresholds of high and/or low activity levels, given their baseline information, and their longitudinal variability, the time window for conversion to MCI was predicted.

Motivated by those mentioned above, the replicability of the results was evaluated. The sample size for a 4-year clinical trial of progression to AD was estimated by defining three outcome measures, namely: (1) the observed longitudinal in-home data, (2) the likelihood of experiencing deviations based on subject-specific thresholds, and (3) annually performed clinical neuropsychiatric assessment^183^.

Learning temporal patterns in sequential data requires: (1) the detection of the onset of change, (2) deciding which information to keep and update, and which information is of the least significance to the meaning change, and lastly, (3) updating the models to account for the detected change for future predictions. To this end, the adoption of sliding windows along longitudinal data, from which the information of least importance is forgotten according to specific rules^184^. These windows are adaptive such that the size of the window grows when no changes are detected and shrinks otherwise for rigorous analysis of the change. Furthermore, among the most reliable models of sequential data are Hidden Markov Models that have been utilized for Daily Activity Recognition^185^. The main merit of such models is that they follow the Markov process, which states that the prediction of the future can be solely based on the present.

Alternatively, reinforcement learning has been proposed for the prediction of the yearly progression of AD whereby the reward was an optimization objective function resulting in defining the differential relationships among the factors and their evolution pertinent to AD progression^186^. Despite the enthusiastic accumulation of dynamic behavioral analysis, how the parallel dynamic measures inter-relate is yet to be elucidated for a complete prediction of neurodegenerative disease patients’ well-being^187^.

## Toward explainable digital phenotyping

### Tackling complexity

Most ageing functional decline processes, especially those attributed to neurodegeneration, are nonlinear in nature^188^. The complexity of the impaired brain is a barrier to understanding fine-grained neurodegenerative manifestations. To learn this complexity, selective and recurrent analyses need to be applied to behavioral data, stemming from recent trends in systems neuroscience^189^. From the data analysis perspective, frequent confusion is that of circularity that arises from the assumptions made to selectively analyze behavioral data, which is a composite of true effect and noise. To avoid distorted results and invalid statistical inference due to selective analysis, the selection criteria among the noisy data should be statistically independent of the analysis results, in addition to dividing the dataset into training, testing and validation subsets^190^. In addition to modeling uncertainty, this characteristic should be accounted for when designing decision-making systems in neurodegenerative diseases medicine. Moreover, the complexity arises from the multifaceted symptoms present in big data. A complex system, in this context, refers to that of multiple interacting aspects with an unpredictable outcome, which renders studying one aspect in isolation from others insufficient^191^. One way to address this is through multidimensional data analysis and graph-based network approaches^192^. To account for the biological consequences on the digital phenotype, smartphone data were integrated unprecedentedly with metabolic measures in real time^193^. This way, the impact of lifestyle habits, such as diet, physical activity, sleep, smoking, and exposure to toxics, can be monitored via metabolic phenotyping alongside behavioral attributes, achieved via longitudinal and simultaneous urine and digital data collection.

In AI-supported decision making, the quantification of uncertainty is often neglected, albeit it can lead to more principled outcomes^194^. To this endeavor, medical ML platforms should avoid reporting a prediction if high-level uncertainty is present, by seeking additional human expertise, a paradigm known as human-in-the-loop, or additional data. The sources of uncertainty include, but are not limited to, data noise which contributes to aleatoric uncertainty, model parameters and model selection resembling epistemic uncertainty or even missing information and bias. Just as a complex patients’ representation will cause uncertainty in the physician’s room, a point in the test dataset far from any point in the training dataset is a source of uncertainty. Techniques of uncertainty quantification are heavily linked to the type of employed models. One popular way to quantitatively represent predictive uncertainty is by the 95% confidence interval^195^. Alternatively, for deep learning, the methods usually used to estimate uncertainty include Monte Carlo Dropout^196^ and ensemble models^197^. The former stems from Bayesian variational inference, while the latter refers to combining the predictions of multiple networks trained on the same dataset.

**Fig. 4:**
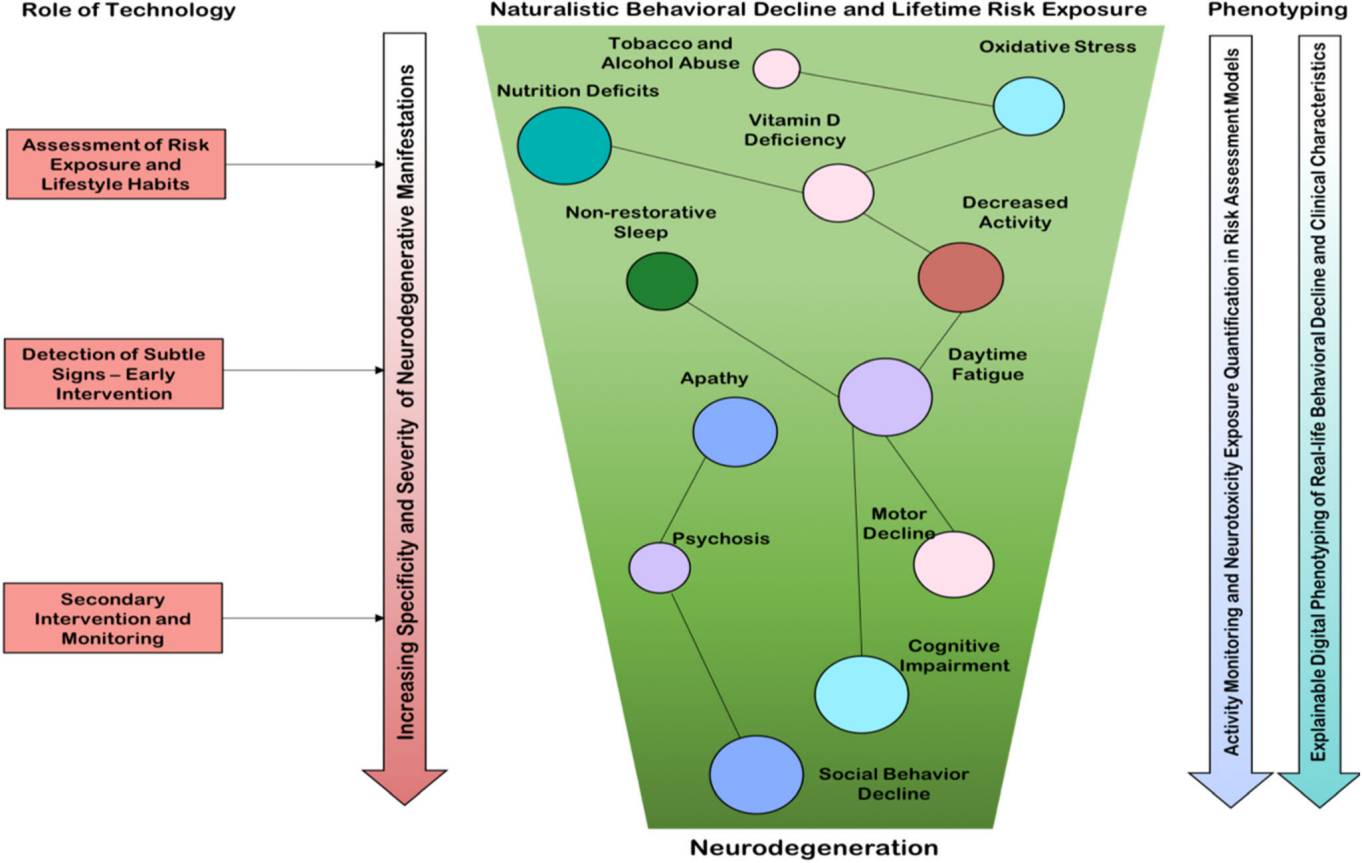
The causality model of real-life neurotoxicity exposure and neurodegenerative manifestations. An ideal risk assessment model starts in early adulthood, where specific lifestyle behaviors predisposes the brain to neurodegeneration, but without developing symptoms. With aging, when disease tolerance starts declining, neurodegenerative manifestations begin as subtle behavioral decline in-the-wild, affecting motor, cognitive, and emotional domains. The role of digital technology in characterizing the phenotype can be viewed as (1) toxicity exposure and risk assessment and (2) explanations for the quantitative behavioral decline.

Another feature of particular importance but a relatively low emphasis on neurodegeneration is causality. The century-old debate that neurodegeneration starts well before diagnosis affirms the dire need to change the medical landscape with predictive paradigms^198^. Therefore, an accurate definition of causality implies that the consequence (neurodegeneration) happens after and is the outcome of the risk factor. However, it is also well conceived that the interplay of genetics and environmental factors is the main contributor to neurodegenerative phenotype modulation^199,200^. For this purpose, modeling causality in neurodegeneration, by accounting for neurotoxicity exposures and lifestyle habits and developing risk assessment models have been facilitated by progress in computational modeling and AI (see Fig. 4), but should ideally begin in early adulthood, given the undefined time duration between exposure and disease onset. Of particular interest in this realm is the role of environmental factors with an apparent connection to neurodegeneration that can be measured and analyzed for reliable predictive models^201^. Among these factors is the role of pesticide exposure^202^, dietary habits^203^, tobacco smoking^204^ and other neurotoxic materials that stimulate oxidative stress and trigger the deposition of neurofibrillary tangles.

Even at the cellular level, genetic predisposition and environmental stressors result in the selective failure of particular populations of neurons, which is, in turn, a mechanism linked to the heterogeneous manifestations of neurodegeneration^205^. This implies that multiple stressors interact and reinforce each other to initiate multi-level chronic perturbation that leads to neurodegeneration and that this can start at any age. The resulting neural dysfunction, however, is aggravated in the elderly, when age-related stressor tolerance fails to withstand the load of the disease and at this point, the symptoms begin to appear. Stemming from here, modeling causality in neurodegeneration entails aggregating lifestyle data from at least early adulthood. Given, for instance, the protective role of physical activity against cognitive decline and dementia^206^ it is not only employed as a predictor for neurodegeneration but also as a modality to reinforce behavioral change. Taken together, we propose a multifaceted trajectory model and the distributed role of digital technology in (1) the quantification of risk exposure and lifestyle habits and their relationship to neurodegeneration, (2) the detection of early subtle signs, and (3) the management of symptoms and disease progression at later stages, as illustrated in Fig. 4.

What we need is decision-based systems with human reasoning power. To capture knowledge about the complex and dynamic properties of the systems with close functionality to human reasoning, fuzzy cognitive maps (FCMs) were explored, depending on real, temporal patient data. The graphical representation of causes and effects via connected nodes, yields a simple and symbolic description of systems behavior, employing the accumulated knowledge of experts^207^. For PD, for instance, FCMs, combined with nonlinear Hebbian learning, result in a diagnosis decision process closer to that of experts^208^. This section reviews the methodological principles of decision-making systems that account for complex neurodegenerative manifestations.

Another source of complexity that arises when naturalistic data are processed in-the-wild is the context attributes that are undetachable from the behavioral patterns^209^. Consequently, for accurate interpretation of the data, the aim is to define context-enriched behavioral patterns^210^. To this end, contextual information has been accounted for in behavioral data mining by multiple approaches, including (1) accounting for contextual information as features in the model, (2) incorporating contextual information in the preprocessing stage to enhance granularity as in probabilistic graphical models^211^, and (3) the detection of anomalies by fusing the knowledge from multiple context domains^212^. To infer high-level information from contextual patterns, frequent data mining^213^ and complex event processing^214^ were combined with learning personalized daily habits and identifying contextualized behavioral change^215^. Table 2 summarizes the characteristics of neurodegeneration and the corresponding modeling paradigms.

**Table 2:** Neurodegenerative behavior characteristics, their clinical definition, modeling characteristics and corresponding relevant references.

Behavioral characteristic	Clinical definition	Modeling characteristics	Reference(s)
Heterogeneity	The heterogeneity of neurodegenerative manifestations stems from different characteristics of the aggregated proteins and the affected brain structures as well as the spatial gradient of gene-expression pathways and inherent heterogeneity of neurons and their synaptic configuration	• Linear mixed-effects models • Long-short-term memory followed by dynamic time warping framework • Co-pathology clusters definition • Gaussian Bayesian networks (GBN)	^280–285^
Temporality	The progression along the prodromal phase, and the progression along the disease itself, including treatment response, the progression throughout the stages of the disease, i.e., increase/decrease of severity, transformation points	• Self-supervised neural networks • Multivariate mixed-effect models • Recurrent neural networks • Bayesian networks • Reinforcement learning	^185,186,286,287^
Complexity	The multi-level disease mechanisms. The inter-relationships between the genetic, molecular and neural factors behind neurodegeneration is uncertain in nature	• Graph-based network approaches • Fuzzy cognitive maps • Probabilistic Bayesian networks • Ensemble models • Monte Carlo dropout methods for uncertainty in AI	^65,192,194,195,288,289^

### An update on “black-box models”

**Fig. 5:**
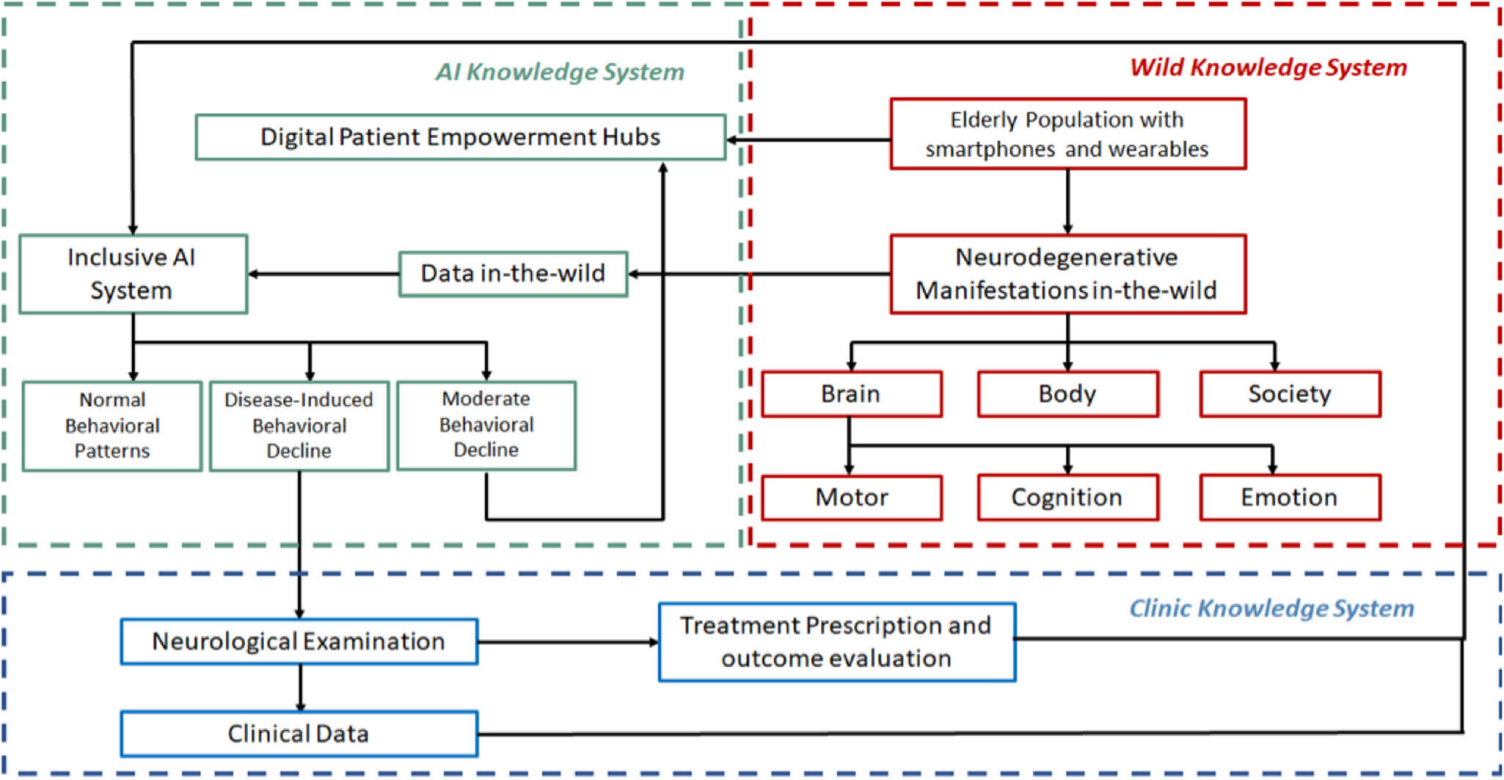
The flow of knowledge in a multidisciplinary co-creation approach. The AI Knowledge System is centered on data acquired in-the-wild and digested by novel machine/deep learning algorithms to infer disease-induced behavioral decline. The Wild Knowledge System that includes elderly populations who interact with smartphones and wearables resulting in a rich space of behavioral data reflective of early neurodegeneration. The Clinic Knowledge System that involves medical decisions based on neurologists evaluation, generating clinically-validated multimodal datasets of early neurodegeneration.

Deep learning needs neuroscience, literally. By mimicking how the brain learns, artificial neural networks are set at the center of efficient “big-data” analysis by tweaking the strength of connections between multi-layer neurons during training, ultimately leading to robust classification and/or regression performance^216^. Nevertheless, the information flow within the network remains elusive, particularly problematic when neural networks are employed to recognize disease markers and provide a diagnostic or prognostic decision. As a consequence, we argue that neural networks should ultimately foster the delivery of meaningful patient-specific knowledge based on explanations that are perceivable by neurologists and neuroscientists, resulting in mutual knowledge exchange (see Fig. 5). Away from the cumbersome heatmaps and salient features in XAI^16^ that leave us with a tradeoff between simplicity and completeness^217^, we focus on drawing interpretations given the character of the input data and the nature of the models to transform the AI systems previously conceived as “black-box” to knowledge-based systems. In the explainability paradigm, the top-performing algorithms of deep learning should be a priority, given their superior performance in computational predictions. From a methodological perspective, deep learning recently outperformed conventional ML models, but their role in modeling neurodegenerative data has been neglected. Predictions are multivariate; the algorithms predict by relatively weighting and combining multiple factors to obtain a risk or probability^218^. Furthermore, according to the statement of Transparent Reporting for a multivariate prediction model for Individual Prognosis or Diagnosis (TRIPOD), developers of predictive models should ensure that besides complete reporting of model performance on testing and validation datasets, their models can be studied further and generalized to fit new databases, thereby supporting reproducibility^219^. In addition, to create personalized knowledge-based systems, integrating human intelligence (HI) and AI has been foreseen as a promising avenue, especially when neurodegeneration’s interacting risk factors are considered^220^. Adding human reasoning power, which is based on neurologists’ experience to the computationally rigorous AI algorithms, is not explored yet, but multidisciplinary work is essential.

In domains with high uncertainty^221^, Bayesian networks offer an appropriate interpretability paradigm based on the probability theory^222^. In other words, Bayesian networks represent the decision-making system components as independent nodes and their conditional probabilities in a network structure, constituting an expert knowledge inference paradigm. Among its applications are the progression prediction of MCI to AD^223^ and the evaluation of the severity of bradykinesia and fine motor impairment in PD^224^. Although the challenges of large, labeled data and data noise in-the-wild are discussed elsewhere, the current paradigms toward self-supervised deep learning models and transfer learning are foreseen to mitigate them^225^. We particularly show how neuroscience-inspired deep learning rules facilitate the advancement of digital technology in neurodegenerative disorders^226^.

Another aspect of mitigating the “black-box” nature of AI in medicine is the call for transparency^227^ and the consideration of ethics, especially as AI is being integrated into the clinical workflow^228^. Despite the pressing need for this, a framework for managing the transparency of AI systems developed for neurodegenerative behavior in-the-wild is not formulated yet. To establish those above and foster knowledge production based on fruitful data, a multidisciplinary group of researchers and funding agencies gathered to jointly construct the so-called FAIR Principles^229^. Besides digital data collection, labeling and storage, they advocated for data stewardship, which refers to long-term data curation. They are re-used for downstream investigations, in isolation or combined with newly collected datasets. For this FAIR endeavor, four pillars were emphasized: findability, accessibility, interoperability and reusability. These principles should encapsulate not only the data, but also the algorithms and the validation platforms, resulting in reproducible analytical pipelines. Within the milieu of behavioral data in-the-wild, reproducibility should be reinforced so that data analysis leads to consistent predictions and interpretations to support innovation and knowledge discovery^230^. Specifically, behavioral data require rich metadata that explain the context, quality and conditions under which they are collected, thereby reinforcing the reliability of the analysis outcome. Furthermore, while AI proved beneficial in detecting and managing early neurodegenerative phenotypes, Rubeis^231^ defined four main risks that, according to him, are deemed the disruptive power of AI, namely the dehumanization of care as more emphasis is given to the machines, the discrimination of minority groups who are not represented in the data, the depersonalization of decisions due to the algorithms, the disciplinarian of users through continuous monitoring and data collection. To overcome these risks, ethical AI provides a guiding framework encompassing four main characteristics of medical ethics^232^. These include (1) establishing and reinforcing a common aim in AI (e.g., increasing the interpretability power of subtle behavioral decline in neurodegeneration), (2) defining the norms of professionalism and good conduct in AI by cultivating a shared professional culture across the variable medical specialties, (3) developing methods to translate research outcomes to clinical practice as attempted in this review, and lastly (4) enhancing the legal accountability.

Given the potential and attractiveness of digital phenotyping, it is important to consider ethical and legal regulations to avoid unintended consequences associated with privacy and data security^233^. The ethical landscape associated with digital phenotyping data protection varies depending on the usage domain and the relevant stakeholders. For example, data for neurologist-mediated usage are covered by the Health Insurance Portability and Accountability Act. This domain is concerned with informing patients on when and why to use the broadly generated data in the clinical environment^234^. On the other hand, for data scientists’ usage, a new realization of the Institutional Review Board approvals to cover the ethical regulation of digital technology data has been formulated^235^ and for disclosing the rational decisions of the deep predictive models.

Besides that, protecting users’ data is particularly essential as these data sources contain highly granular text messages, emails, and location messages, to name a few. It is not only due to the sensitivity of the behavioral data, but also the impact of the predictions based on the data on employment, litigation, social and other contexts. These regulations need to be disclosed in the informed consent as individuals need to know when, why and how their data will be collected^236^. Moreover, if the digital data are to be incorporated into patients’ EHRs, the patients should be informed that their data will be accessed by third-party (researchers, data analysts, etc.). Therefore, methods to ensure accountability and transparency in such regulations must be implemented. Such legal frameworks will reinforce the trust from the perspectives of subjects enrolled in the digital phenotyping enterprise and preclude misuse of data^237^. Subsequently, data storage and encryption are the core of the ethical perspective. Especially when it comes to data collection, encryption and anonymization of users’ data is the essence of privacy protection^238^. The data are then re-encrypted to be stored in secured cloud servers. In particular, for smartphone-based digital phenotyping, encryption keys are provided to the smartphone application by which the data is encrypted and only the storage server can decrypt the data. Thus, the data cannot be accessed except by the authorized study server during collection, storage and transmission.

### “Value co-creation” in lifestyle medicine

To avoid the dehumanization pitfall of AI, the development of the systems should satisfy the participatory technology design^239^. This process should ideally start with identifying patients’ needs, followed by evaluating the design outcomes. Comprehensive evaluation and appraisal of the prerequisites of a multidisciplinary effort to enhance symptom-specific neurodegenerative disease care are yet to be conducted. This is centered on a value co-creation approach^240^, whereby the designers, developers, bioengineers, neuroscientists, neurologists and end-users (i.e., patients) exchange knowledge with equal power distribution in an innovation process to optimize design specifications for better diagnostic and therapeutic yield^241^. In neurodegeneration, co-creation refers to the generation of unified interpretations and meaningful multidimensional descriptions of the naturalistic behavior^242^, and brings together desperate mechanistic characteristics of neurodegeneration inherent to community-dwelling elderly. This type of explanation should ideally be co-created, and the lack of co-creation frameworks still hinders precise behavioral identification models that aid in personalized neurodegeneration detection. To foster such a framework, identifying tools to engage the relevant stakeholders for the generation of ideas, their implementation and dissemination is essential^243^. The ideal developmental process starts with assessing patients’ needs, identifying personalized technological characteristics, setting up data management protocols, and ends with a quality of care assessment. More specifically, to overcome the current pitfalls of patient-centered medicine, a systemic approach that encompasses internal knowledge production enabled by data-driven computational paradigms and external coordination of between-institution networks is essential^244^. In fact, besides the unprecedented growth of digital technology, we need a more humanistic perspective, whereby the patients’ role in healthcare awareness and compliance to medical intervention is centralized, as in the Diagnosis, Access, Risk assessment and Transparency (DART) model^245,246^. In other words, the patients should not only be viewed as the “data source” during the innovation process but also as an empowered member who influences the outcomes of the innovative product^247^. Central to this aim, patient characteristics and educational level (e.g., illiteracy, technological fluency) and concepts adopted from social gerontology should be considered when neurodegenerative disorders are the point of interest.

Especially in assistive technology, the ageing body is always viewed as a malfunctioning machine. We argue that the ageing population is highly heterogeneous, multidimensional, and nonlinear, and sets between different strengths and weaknesses, thereby requiring a breakthrough in design personalization. Without taking the points above into account, along with the centralized role of the patient in this multidisciplinary landscape, the digital biomarker industry will remain in great jeopardy. However, a problem that arises here is the self-awareness and the stigma associated with the end-users experiencing a behavioral decline. For the elderly in particular, the degradation of metacognition, that is, self-knowledge of the declining cognitive and functional capacity^248^, compels as a barrier to the positive impact of home-based digital technology and the way it reshapes their quality of life.

We also eventually note potential pitfalls, such as the lack of RCTs assessing the interventional efficiency of AI technology, as the latter should be perceived as a standard medical intervention. We also urgently call for a global and multidisciplinary consortium to ensure the proportionate delivery of neuro-technology to low-income populations to mitigate the dilemma of unrepresented populations in AI algorithms.

Recent research trends document a substantial growth in the use of connected digital devices in clinical research, with a compound annual growth of ~34% between 2000 and 2017^249^. In particular, for patients, digital technology increases enrollment in clinical trials, given the unobtrusive nature of data collection. Thus, the concept of “digital clinical trials”^250^ has the potential to enhance patients’ engagement and define target outcomes and reduce clinical trial cost. Consequently, real-world initiatives emerged as examples reflecting the feasibility and potentiality of digital clinical trials. For example, the i-PROGNOSIS European Union-funded Horizon 2020 project offers a real-world example of PD patients scenarios undergoing digitized monitoring and intervention through a co-created smartphone application^251^. This initiative involved academics, neurologists and engineers from Greece, Belgium, Germany, the United Kingdom (UK), the United Arab Emirates (UAE) and the United States (US), and collected data passively from 2350 volunteers, from which 373 were self-reported PD patients. The holistic nature of such digital enterprise can be viewed from diagnostic and interventional perspectives. For the former, the smartphone application facilitates the acquisition of typing kinetic parameters and/or how the subject holds the mobile phone to infer fine-grained mobility parameters related to bradykinesia, tremor and akinesia.

Regarding PD intervention, the i-PROGNOSIS initiative formulated a personalized Serious Game Suite that combines motivational and rehabilitation elements to reinforce PD behavioral change associated with motor, emotional and cognitive facets^252^. The games are adaptive to each patient’s profile through AI algorithms that analyze the performance of patients longitudinally. On the other hand, to set forward the facets for impactful remote AD functional assessment and management, the processes by which relevant functional domains are selected and relevant stakeholders are engaged^253^. Especially in AD, activities of daily life involve a range of cognitive and executive functions associated with prognosis and hence, offer promising remote measurement targets. A pragmatic example tool is the Altoida Digital Neuro Signature (DNS), which uses a battery of augmented-based tests for eye movement, hand dexterity, gait and cognitive functions delivered by a tablet application^254^. The DNS proved more sensitive to executive function changes than conventional neuropsychiatric assessment scales.

## Clinical implications

Our work has important implications for clinical practice. First, the future should be devoted to exploring the commonality, complementarity and conflict between neurodegenerative manifestations in the digital and the biological domain^255^, as we have attempted in this review. Instead of the shadowing effect digital data currently has in clinical practice, the explanations drawn from them and the background clinical data should create a sort of “data doubles”^256^. In this way, the abstracted “data-double” becomes materially forceful by identifying those at-risk and empowering them, thereby becoming a closed-loop system that generates patient-specific knowledge, updating the user with them and imposing personalized behavioral enhancement strategies^257,258^. Currently, the validation of the digital biomarkers lags well behind the conventional clinical tests, behind which decades of validation effort were spent^259^. Our purpose with this review was to initiate the validity paradigm for digital behavioral data across the heterogeneous populations of neurodegenerative diseases and to convey the explanatory power by analyzing the variance of behavioral attributes. This validity is, however, unlikely to be materialized without large-scale controlled clinical trials with regulatory approvals. For example, smartphone-based assessment of motor decline in PD was incorporated in phase 1 clinical trial for the first time in ref. ^260^ and conveyed the clinical validity of digital biomarkers, but lasted only 6 months for 45 PD patients.

Another rate-limiting factor behind the pitfalls of early detection and prevention of neurodegeneration is the lack of our understanding of its causal mechanisms. The fact that tau pathology that triggers the onset of neurodegeneration is associated with multifaceted behavioral change^261,262^ carries important implications in the context of precision medicine. A plausible solution lies in the aggregation of latent variables in efficient computational models attempted by all relevant stakeholders, thereby accounting for the knowledge that flows from multidisciplinary interaction that is transformed into a more comprehensible one. The high variance inferred from naturalistic behavior in-the-wild confirms the in vivo evidence of the selective anatomical pathways of neural degeneration and the concept of co-pathology in neurodegeneration that is not accounted for yet. In the competitive space of biomarker discovery, the accumulated research effort needs to generate decision-making tools with sufficient technical and biological validation to allow them to be confidently used in clinical practice as in drug discovery^263^. The focus in the future should be on formulating databases that are representative of the whole population. The problem of unrepresented populations can only be overcome if the current biomarkers consortium works to mitigate the disproportionate delivery of the technology to previously untargeted populations.

The interest in involving digital biomarkers in clinical trials has recently increased but still requires regulatory effort^264^. However, the impedance toward this endeavor lies in the need for a large sample size and the daunting organization cost. However, increasing the frequency of data collection is foreseen to offset the need for a “big population pool”. Moreover, accounting for serendipitous scientific and medical questions within the digital enterprise of Research and Development (R&D) is important for neurodegenerative diseases^265^. An example of such a need lies in symptom interplay in neurodegeneration.

We foresee a future for digital biomarkers similar to imaging biomarkers in neurology^266,267^. A daunting need for benchmarking databases exists, such as the Alzheimer’s disease Neuroimaging (ADNI) database and long-term clinical trials that prove their reproducibility and replicability. In this vein, the collaborative work between clinical practice and empirical research becomes essential to define accurate thresholds of what is considered normal and what is not. Moreover, the analysis of complicated cases and the characterization of their short- and long-term behavioral characteristics should be a priority, given that these types of cases have been excluded from the studies, perhaps leading to biased conclusions on biomarkers reliability. Another question is what expectations clinicians can expect in their everyday practice given the paradigm of explainable digital phenotyping^268^. For instance, it is also important to note the actions that should be taken when clinical and technological results do not match.

The dilemma of missing data is particularly important when designing longitudinal behavioral analysis systems, impeding the efficient execution of the intricate computational power. Despite the previously claimed suitability of applying Bayesian networks to incomplete datasets, optimizing the network weights of the data variables remains a challenge^269^. For the prediction of MCI progression, for instance, missing data were first estimated using mutual information between two features and Newton interpolation algorithms to adjust for missing data before a Bayesian network on the feature ordering is used to estimate the posterior^269^. Nonetheless, using such models for a set of more than two variables render us with a tradeoff between complexity and completeness. The algorithms, not the data, will be proven transformative^270^.

The brain is a dynamic organ, subject to continuous changes in its structure and function from the molecular to the network level^154^. Self-generated and environmental inputs influence this plasticity, and its output is propagated to microscopic, mesoscopic and macroscopic tiers (the focus of the current review). By reinforcing design-optimized environmental rehabilitation strategies, we propose that neuroplasticity could be the route to enhanced brain functions, particularly during early disease stages. This, however, comes after identifying the behavioral deficits discussed in this review. More importantly, analyzing the behavioral parameterization specific to every individual/patient is essential for delivering personalized, adaptive therapeutic strategies. Decoding the subject-level variability will inform the emotional, cognitive and sensory-motor characteristics and, subsequently, the preferred rehabilitation strategy, task characteristics and its degrees of freedom. We foresee that this paradigm will lead to a completely different landscape for healthcare practice in neurodegeneration, which is predictive, personalized and participatory.

Neurodegenerative disease patients’ care is going to change dramatically in the coming years through an evolutionary process driven by (1) the nationwide implementation of EHRs and (2) the taxonomy of explainable digital phenotyping through which the neurodegenerative manifestations are disentangled^271^. The dynamic, technology-driven development of medical decision making led to a huge spectrum of interactive health data, engaging patients and healthcare professionals in PD care. In turn, with the worldwide digital advancement, it is foreseen that most countries’ provision is currently toward innovative EHRs.

Nevertheless, such technology-based measures still need to be utilized in clinical trials, necessitating future efforts toward validating measurement outcomes, standardizing clinically relevant biomarkers and establishing an integrated platform for clinical and digital data^272^. Integrating the two parallel worlds, namely evidence-based medicine and real-life personalized phenotypic characteristics, defines a new ecosystem of patient-centered clinical pathway^273^. This paradigm shift, where patients are empowered to move in the center of medical decisions, requires the definition of a new Digital Clinical Pathway (DCP) that considers multidisciplinary perspectives. In contrast to classical clinical guidelines, this DCP will infer mobile health measurements and merge the “big medical data” and healthcare professional resources into a new, hybrid workflow. Within the DCP, the data records for specific treatments are optimized, stored and transmitted to all the stakeholders of this “Co-Creation” approach. In particular, through deep analytic algorithms driven by AI, the decisions reflect a new realization of “connected care” of neurodegenerative diseases. The essence of this paradigm will be the multimodal information inferred from clinical records and contextualized real-life data.

Accordingly, future research endeavors include advancing explainable deep learning models to arrive at personalized inference models. Another important aspect will be reporting the target parameters, including treatment options, to both clinicians and patients.

More importantly, deriving disease subtypes based on EHR data is key in deeply personalized phenotyping of neurodegenerative diseases. Data-driven disentanglement of neurodegenerative heterogeneity in PD and AD through deep representation learning from millions of EHRs has been possible^274^. In particular, EHR-derived information, such as genetics, medications, clinical notes and laboratory results, is used in dimensionality reduction deep learning methods such as convolutional autoencoders, word embeddings and convolutional neural networks to infer low-dimensional vector representations of patients. The resulting vectors are eventually hierarchically clustered into multi-disease and disease subtype groups. For instance, PD stratification analysis results in two main subgroups: motor (tremor-dominant) and non-motor subgroups. Similarly, AD stratification analysis results in three subgroups based on AD onset, disease progression and severity of cognitive impairment. Nevertheless, there are significant limitations associated with stratification analysis, including noisy and unlabeled data, hospital-specific biases related to the inherent structure of EHRs and the choice of the complex disease traits that play a role in the analysis outcome. Data engineering experts are yet to reach the equilibrium between hand-engineered patient features and end-to-end deep learning models and to ensure the generalizability of the proposed models.

## Conclusion

To conclude, neuroscience is an interdisciplinary domain that affords cooperation between important academic domains that still need to be improved by methodological gaps^275^. In this review, we attempted to reinforce the explanatory power of subtle neurodegenerative behavioral decline in-the-wild through the explainable digital phenotyping taxonomy that delineates borders between neuroscience, neurodegenerative diseases, healthcare and AI. This taxonomy rests on the ecological transition of neurodegeneration characteristics whereby the patients constitute the microsystem of the taxonomy, and their interaction with the environment and their lifestyle context resembles the mesosystem. Besides that, we defined the macrosystem of the proposed taxonomy as the network connecting neuroscientists, neurodegenerative diseases healthcare professionals and bioengineers who are the stakeholders of the co-creation of new-generation AI-supported nosology for precise behavioral interpretation.

## Supplementary information

Supplementary Table 1

## Data Availability

Not applicable. Any data regarding the search strategy can be directed to the corresponding author.
