# Supplementary material for: A scoping review of neurodegenerative manifestations in explainable digital phenotyping
Source: NPJ Parkinsons Dis. 2023 Mar 30;9:49. doi: 10.1038/s41531-023-00494-0 (PMC10063633; doi:10.1038/s41531-023-00494-0)
Supplement: Supplementary file 1 — Supplementary Table 1 [file 41531_2023_494_MOESM1_ESM.pdf]

Supplementary Table 1: Systematic Analysis of the Included Studies

**Author, Year of Publication**	**Neurodegenerative Disease (PD/AD)**	**Neurodegenerative Symptom (Tier Level)**	**Measured behavior**	**Clinical Utility and Outcome**
Iakovakis et al. (2020) [224]	PD	Motor Function (Fine motor decline)	Keystroke dynamics Upon Smartphone Typing	Estimation of fine motor impairment index and tremor.
Papadopoulos et al. (2020) [54]	PD	Motor Function (Fine motor impairment and tremor)	Keystroke dynamics and Accelerometer data	Fine motor Impairment and Tremor Indexes Estimation
Ntracha et al. (2020) [57]	AD	Cognitive Function (Linguistic decline)	Spontaneous written speech texts	NLP and deep learning for linguistic feature analysis
Iakovakis et al. (2018) [51]	PD	Motor Function (Fine motor decline)	Keystroke dynamics Upon Smartphone Typing	Early PD prediction
Prince et al. (2018) [43]	PD	Motor Function (Bradykinesia)	Multimodal task-specific measures obtained via application	Early PD prediction using multimodal analysis
Rusz et al. (2021) [61]	PD	Motor Function (Speech Apraxia)	Acoustic features extracted from speech	Detection of iRBD-PD given acoustic feature
Laganas et al. (2021) [62]	PD	Motor Function (Speech Apraxia)	Speech collected during Phone call	Early PD detection from running speech
Lonini et al. (2018) [64]	PD	Motor Function (Upper Extremity Bradykinesia)	Home-based IMU sensors	Evaluation of upper extremity motion during activities of daily life.
Nahar et al. (2020) [290]	PD	Motor Function (Fall Prediction)	IMU sensors	Fall Prediction deep learning model
Ellis et al. (2015) [69]	PD	Motor Function (Gait Variability Analysis)	IMU sensors	Early PD prediction
Bikias et al. (2021) [70]	PD	Motor Function (Gait parameters)	IMU sensors	Freezing of Gait detection.
Vergara-Diaz et al. (2021) [72]	PD	Motor Function (Upper extremity measures)	IMU sensors and accelerometer	ON-OFF medication response in PD
Gielis et al. (2021) [77]	AD	Cognitive Function	Klondike Solitaire game scores	Assessment of Executive function
Seo et al. (2017) [78]	AD	Cognitive Function	Kinematic movements during Activities of daily life	VR-based assessment of cognitive skills

Chlasta et al. (2021) [81]	AD	Cognitive Function (Aphasia)		
Beltrami et al. (2018) [82]	AD	Cognitive Function (Aphasia)	Spontaneous Speech	NLP
Ilias et al. (2022) [84]	AD	Cognitive Function (Aphasia)	Multimodal texts and Speech.	Early AD detection from linguistic features
Weizenbuam et al. (2021) [86]	PD	Cognitive Function	Home-based Ecological Momentary Assessment (EMA-self-reports)	Working memory and executive function evaluation
Kubaik et al. (2019)[101]		Emotions (Apathy)	Ecological Momentary Assessment	
Konig et al. (2019) [102]	AD	Emotions (Apathy)	Voice Features	Detection of apathetic AD patients
Saboo et al. (2022) [186]	AD	Cognitive Function	Self-reported data	Reinforcement learning for AD progression prediction
Bulling et al. (2014) [185]		Motor Function	Body-worn IMU	Longitudinal analysis of motor behavior
Kaye et al. (2018) [181]	AD	Cognitive Function	Multimodal data including accelerometer, home-based sensors (cameras) and scheduled self-reports	Longitudinal analysis of executive function
Rusz et al. (2019) [156]	PD	Motor and Cognitive Functions	Speech impairment characteristics	Classification of Multiple System Atrophy parkinsonian and cerebellar subtypes (MSA-P, MSA-C) from PD
Zhang et al. (2019) [157]	PD	Motor and Cognitive Functions	The PPMI multimodal data	Severity classification of PD progression
Malaty et al. (2021) [161]	PD	Motor and Cognitive Functions as a medication response	Multimodal clinical and self-reported data	Identification of the 5-2-1 PD subtype.
Antonini et al. (2021) [162]	PD	Motor and Cognitive Functions as a medication response	Multimodal clinical and self-reported data	Identification of Patients with severe PD Off states.
